# Remission induction in refractory, drug resistant pediatric *PICALM::MLLT10*+ B-cell acute lymphoblastic leukemia by venetoclax

**DOI:** 10.1038/s41375-025-02591-w

**Published:** 2025-04-15

**Authors:** Alexandra Niedermayer, Jana Stursberg, Anke Katharina Bergmann, Martin Zimmermann, Gunnar Cario, Monika Brüggemann, Rolf Köhler, Daniel Steinbach, Christian Reimann, Felix Seyfried, Lüder Hinrich Meyer, Klaus-Michael Debatin

**Affiliations:** 1 Department of Pediatrics and Adolescent Medicine, Ulm University Medical Center, Ulm, Germany; 2 German Center for Child and Adolescent Health (DZKJ), partner site Ulm, Ulm, Germany; 3 Institute of Human Genetics, Hannover Medical School, Hannover, Germany; 4 Clinical Genetics and Genomic Medicine, University Hospital Würzburg, Würzburg, Germany; 5 Department of Pediatric Hematology/Oncology, Hannover Medical School, Hannover, Germany; 6 Clinic for Pediatric Oncology and Rheumatology (Children and Adolescent Medicine I), University Hospital Schleswig-Holstein, Kiel, Germany; 7 Internal Medicine II—Hematology and Oncology, University Hospital Schleswig-Holstein, Kiel, Germany; 8 Institute of Human Genetics, Heidelberg University Hospital, Heidelberg, Germany

**Keywords:** Acute lymphocytic leukaemia, Targeted therapies

## To the Editor

Acute lymphoblastic leukemia (ALL), with over 80% arising from B-cell lineage precursors (BCP-ALL), is the most common hematologic malignancy in children and adolescents. Intensified treatment strategies have increased survival rates to around 90%. ALL induction therapy according to e.g. the AIEOP-BFM ALL 2017 protocol [1] combines high-dose steroids, vincristine, daunorubicin and asparaginase and remission is achieved in most patients after 33 days of treatment, monitored by measurable residual disease (MRD) using clonotypic markers. Subsequent treatment and risk stratification depend on the MRD response and genetic factors. Patients unresponsive to treatment have a very poor prognosis [2].

Here, we report the case of a primary refractory pediatric patient suffering from a *PICALM::MLLT10* + BCP-ALL, who could be salvaged by venetoclax identified by prospective ex vivo drug response profiling (DRP) as the only active drug.

**Fig. 1:**
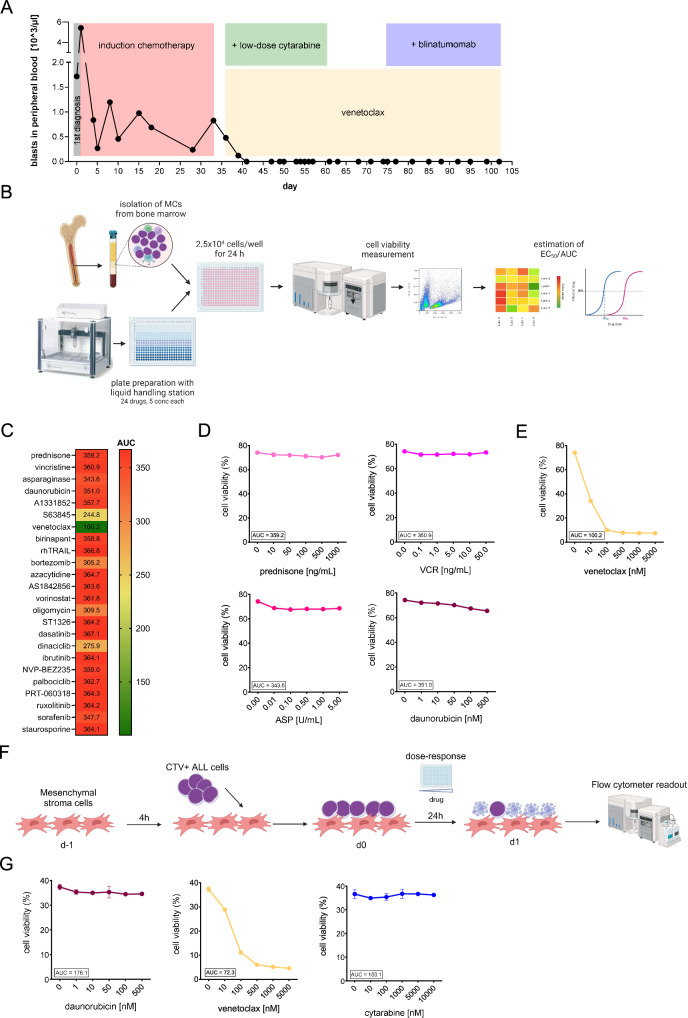
Blast count and drug response profiling (DRP). **A** Blasts in peripheral blood [10^3^/µl]. After primary diagnosis, the patient was treated with induction chemotherapy (prednisone, vincristine, asparaginase, daunorubicin) for 4 weeks. After DRP on day 33, the patient was started on venetoclax (400 mg once daily after a 3-day ramp-up) and low-dose cytarabine (100 mg/m²/24 h for 4 days, followed by 3 cycles of 75 mg/m²/once daily 4 days per week) for 4 weeks. After remission induction, venetoclax was continued and 1 cycle of blinatumomab (15 µg/m^2^/day) was added after 2 weeks of only venetoclax. **B** Schematic overview of the drug response profiling (DRP) performed with mononuclear cells (MCs) isolated from bone marrow on day 33 after induction chemotherapy. DRP plates containing titrations of 24 drugs with 5 concentrations each were prepared using a Liquid Handling Station (LHS) by Brand (Wertheim, Germany). 2.5 × 10^4^ cells/well were incubated with the drug titrations for 24 h and cell viability was measured by flow cytometry (Attune NxT, Thermo Fisher Scientific, Waltham, Massachusetts, USA) using forward/side scatter (FSC/SSC) criteria. Half-maximal effective concentration (EC50) values and area under curve (AUC) were estimated using GraphPad Prism. Created with Biorender.com. **C** Heatmap of AUC estimated for all 24 drugs. AUC was calculated using built-in analysis in GraphPad Prism. The reduction in cell viability ranged from high (AUC = 100.2, indicated in green) to low (AUC = 367.1; indicated in red). High reduction of cell viability indicates sensitivity to the drug (e.g. venetoclax), low or no reduction of cell viability indicates resistance to the drug (e.g. prednisone). **D**, **E** Dose-response curves generated by DRP and AUC of (D) prednisone, vincristine, asparaginase (ASP), daunorubicin and (**E**) venetoclax. **F** Primary bone marrow hTERT-immortalized mesenchymal stroma cells (MSCs) were kindly provided by Dario Campana. For co-culture experiments, MSCs were seeded on day –1 at 5 × 10^3^ per well in 96-well plates in RPMI-1640 medium supplemented with 20% fetal bovine serum, 1% L-Glutamine and 1% Penicillin/Streptomycin. After four hours, 1 × 10^5^ cryopreserved ALL cells from day 33 after induction therapy were stained with 1 µM CellTrace Violet (C34557, Thermo Fisher Scientific) and added to the MSC cultures in technical triplicates. On day 0, samples were exposed to increasing concentrations of the inhibitors for 24 h. Cell viability was measured by flow cytometry (Attune NxT, Thermo Fisher Scientific, Waltham, Massachusetts, USA) using forward/side scatter (FSC/SSC) criteria and CellTrace Violet staining to distinguish ALL cells from MSCs. Data analysis was performed using FlowJo 10.8 software. Created with Biorender.com **G** Dose-response curves generated by DRP using feeder cells and AUC of daunorubicin, venetoclax and cytarabine.

A 10-year-old boy presented with fever of up to 40 °C, fatigue, sore throat and night sweats for about a week, resulting in a Lansky score of 50. Blood test showed mild tricytopenia with 76.5% peripheral lymphoid blasts. Bone marrow (BM) analysis revealed 95% pro-B-ALL blasts (CD19 + , CD22 + , HLA-DR + , CD10-, intermediate CD45), confirming the diagnosis of BCP-ALL without extramedullary disease. An aberrant expression of CD7, CD5, and CD33 indicated a rather immature immunophenotype. After informed consent by the parents, induction therapy was initiated according to the AIEOP-BFM-ALL 2017 protocol (prednisone, vincristine, asparaginase and daunorubicin). Despite initial reduction of peripheral blood (PB) leukemia cells on day 8 (Fig. 1A), assessment by flow MRD on day 15 revealed 70% residual leukemic cells in the BM resulting in stratification to the high-risk group (FCM-MRD on day 15 > 10%). After end of induction (day 33), 92% of blasts in the BM indicated refractory disease with subsequent stratification to hematopoietic stem cell transplantation (HSCT) in case of successful remission induction. While cytogenetic analysis did not reveal high-risk cytogenetic abnormalities, RNA-Seq analysis identified a *PICALM::MLLT10* fusion transcript.

Due to persistence of leukemic cells at day 15 and 33 of induction, we performed an ex vivo DRP using a panel of 24 anti-cancer drugs (Fig. 1B, Supplementary Material) with in vitro concentrations adopted from published, clinically achievable plasma concentrations. DRP confirmed the clinical resistance to prednisone, vincristine, asparaginase and daunorubicin (Fig. 1C, D). However, and in contrast to the overall drug resistance, an exquisite, low-nanomolar activity was identified for the BCL-2 inhibitor venetoclax (Fig. 1C, E).

Based on the intriguing in vitro efficacy of venetoclax in our DRP and its established safety profile in adult and pediatric patients with ALL or lymphoblastic lymphoma [3], we initiated treatment with venetoclax (400 mg once daily). To prevent tumor lysis syndrome (TLS) with a still high leukemic burden in the BM, we performed a 3-day ramp up and observed only a mild increase of phosphate and lactate dehydrogenase, but no severe TLS. Since venetoclax is not an established therapy for this indication, we combined it with low-dose cytarabine (100 mg/m²/24 h for 4 days, followed by 3 cycles of 75 mg/m² once daily, 4 days per week) due to its value in the treatment of acute myeloid leukemia (AML) and the immature immunophenotype of the leukemia cells.

Therapy with venetoclax and low-dose cytarabine resulted in a robust clearance of leukemia cells in PB (Fig. 1A) with morphological and molecular remission in the BM (MRD level <10^4^) after four weeks. Therapy was well tolerated, and hematologic recovery was rapid with granulocyte counts >500/µl after day 20 and no platelet transfusion after day 14 of the individualized treatment.

This successful remission induction was followed by four weeks of consolidation therapy with blinatumomab (15 µg/m^2^/day) and venetoclax leading to MRD negativity with subsequent haploidentical HSCT. Conditioning consisted of total body irradiation and etoposide, followed by post-transplant cyclophosphamide. Six weeks after HSCT, the patient showed complete chimerism (100/100) in both granulocyte and lymphocyte compartments. 14 months post-transplant, the patient remains in remission with a Lansky performance status of 100 at the time point of this report.

Pediatric patients with refractory ALL and induction failure have a very poor prognosis [2]. Our patient did not present any classical high-risk factors (hyperleukocytosis, hypodiploidy or unfavorable genetic alterations according to AIEOP-BFM ALL 2017 study protocol e.g. *BCR::ABL1*-fusion) that would indicate non-response to therapy. However, a *PICALM::MLLT10* fusion was detected, which results from a rare but recurrent t(10;11)(p13;q14-21) chromosomal translocation [4]. This fusion is predominantly detected in immature forms of acute leukemia (AL), often with a mixed T-cell (CD5, CD7) and myeloid phenotype (CD13, CD33), mostly resulting in the classification as T-ALL, AML, acute undifferentiated leukemia (AUL), AL of ambiguous lineage (ALAL) or mixed-phenotype AL (MPAL) [5, 6]. Remarkably, our patient showed a BCP phenotype with aberrant expression of T- and myeloid-markers, which has only been described sporadically in the context of *PICALM::MLLT10* rearranged leukemias [5, 6].

In T-ALL and AML, *PICALM::MLLT10* fusions are associated with a poor prognosis [5–7], e.g. 5-year overall survival rates of only 26% in patients with *PICALM::MLLT10* + AML, which is consistent with the induction failure in our patient.

A most intriguing finding of this a priori refractory leukemia was the exquisite sensitivity to venetoclax ex vivo on the background of an otherwise absolute resistant DRP.

After initiating treatment with venetoclax and low-dose cytarabine, we subsequently performed a miniaturized version of the DRP with cytarabine as it was not part of our standard panel. We used cryopreserved leukemia cells of the patient isolated after induction chemotherapy and performed the assay with the support of feeder cells (hTERT MSC, provided by Dario Campana) due to the poor viability of thawed primary BCP-ALL (Fig. 1F, Supplementary Material) [8]. In line with the DRP performed on fresh cells, cryopreserved leukemia cells showed resistance to daunorubicin and high sensitivity to venetoclax but no sensitivity to cytarabine (Fig. 1G), strengthening the significance of venetoclax in the successful treatment of this patient.

This underscores the potential of DRP as a powerful diagnostic tool in personalized precision medicine to predict in vivo drug responses as also demonstrated in the non-interventional SMARTrial and the VenEx trial [9, 10]. In the VenEx trial ex vivo venetoclax sensitivity was the strongest predictor for a favorable treatment response and survival in patients with AML that were treated with 5-AZA/VEN [10].

Venetoclax displaces pro-apoptotic BAX and BAK from anti-apoptotic BCL-2 to form pores in the outer mitochondrial membrane ultimately leading to cell death induction. Appropriate levels of BAX/BAK bound by high levels of BCL-2 indicate a BCL-2 dependence in the leukemia cell, mediating effective cell death induction upon venetoclax treatment.

**Fig. 2:**
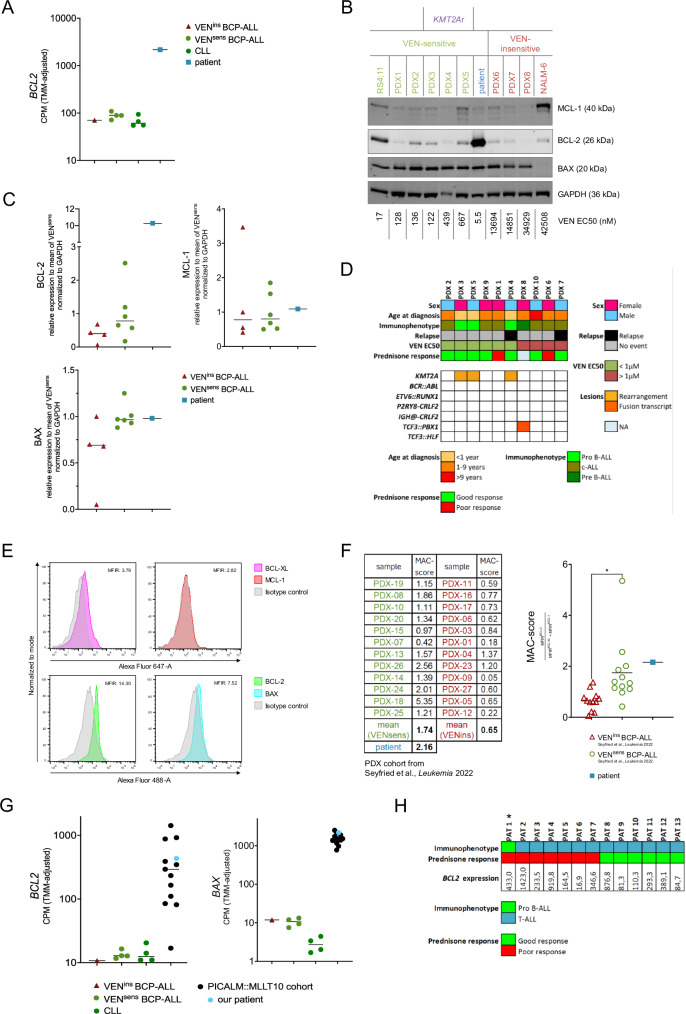
Expression analysis of BCL-2 family proteins. **A** Trimmed Mean of M values adjusted Counts Per Million (TMM adjusted CPM) expression values for *BCL2* (line at median). Venetoclax sensitive (VEN^sens^) samples are defined by EC50 values < 1 µM, venetoclax insensitive (VEN^ins^) samples are defined by EC50 values > 1 µM. The VEN^ins^ samples is PDX 10. VEN^sens^ samples are PDX 3, 4, 5 and 9. **B** Western Blot analysis of protein lysates of VEN^ins^ (*N* = 4), VEN^sens^ (*N* = 5) BCP-ALL samples and of bone marrow aspirate of the patient from day 33 were performed using anti-BCL-2 (#15071, clone 124, Cell Signaling Technology, Danvers, Massachusetts, USA), anti-MCL-1 (#94296, clone D2W9E, Cell Signaling Technology), anti-BAX (#2772, Cell Signaling Technologies) and anti-GAPDH (#ADI-CSA-33S-E, clone 1D4, Enzo, Farmingdale, New York, USA) antibodies; and StarBright Blue 700 goat anti-mouse IgG (#12004158, clone M700) (Bio-Rad Laboratories, Hercules, California, USA) and StarBright Blue 700 goat anti-rabbit IgG (#12004161, clone R700) (Bio-Rad Laboratories) secondary antibody. Immunoblots were developed using fluorescence and densitometric analysis was performed with ImageJ Software. RS4;11 and NALM-6 were purchased from DSMZ (Deutsche Sammlung für Mikroorganismen und Zellkulturen, Germany) and authenticated with STR profiling; mycoplasma contamination was excluded using MycoStrip® (#rep-mysnc-100, Invivogen). PDX samples were previously generated by intravenous transplantation of ALL cells into female NOD/SCID mice (NOD.CB17-Prkdcscid, Charles River) as previously described [8]. EC50 values were determined after 24 h treatment with increasing concentrations of venetoclax (VEN) (0, 10, 100, 500, 1000 and 5000 nM), measuring cell death by flow cytometry using FSC/SSC criteria. Venetoclax sensitive (VEN^sens^) samples are defined by EC50 values < 1 µM, venetoclax insensitive (VEN^ins^) samples are defined by EC50 values > 1 µM. **C** Densitometric analysis of BCL-2, MCL-1 and BAX protein expression was performed using ImageJ Software and values were normalized to GAPDH loading control; line at median. **D** Patient data of the BCP-ALL PDX samples. Prednisone good response (PGR) is defined as <1000 blasts/µL in peripheral blood on day 8 after the 7-day prednisone prephase of ALL induction chemotherapy and prednisone poor response is defined as >/=1,000 blasts/µL respectively. **E** Basal protein levels of the anti-apoptotic proteins BCL-XL, MCL-1 and BCL-2 and of the pro-apoptotic BAX were determined by intracellular FACS staining on cryopreserved leukemia cells isolated on day 33 after induction therapy using rabbit anti-BCL-XL (Alexa Fluor 647, #86387, CST), rabbit anti-MCL-1 (Alexa Fluor 647, #78471, CST), mouse anti-BCL-2 (Alexa Fluor 488, #59422, CST), mouse anti-BAX (Alexa Flour 488, #633604, BioLegend), mouse IgG1 Isotype Control (Alexa Fluor 488, #4878, CST) and rabbit IgG Isotype Control (Alexa Fluor 647, #3452, CST) in triplicates. Representative FACS plots are shown and mean fluorescence intensity ratios (MFIR) of BCL-XL, MCL-1 (Alexa Fluor 647), BCL-2 and BAX (Alexa Fluor 488) normalized to their isotype controls were calculated as mean values from technical triplicates. **F** MFIR of BCL-XL and MCL-1 (Alexa Fluor 647) as well as BCL-2 (Alexa Fluor 488) of a large PDX cohort comprised of VEN^sens^ (*N* = 12) and VEN^ins^ (*N* = 12) samples were extracted from Supplemental Figure 6 of Seyfried et al., *Leukemia* 2022 [8] and used together with the MFIR of BCL-XL, MCL-1 and BCL-2 in our patient to calculate the “mediators of apoptosis combinatorial score” (MAC-score) as previously described by Waclawiczek and colleagues [12]. The MAC-Score was calculated using the following formula: $\frac{{{MFIR}}^{{BCL}-2}}{{{MFIR}}^{{BCL}-{XL}}+{{MFIR}}^{{MCL}-1}}$ MAC-scores of VEN^sens^ and VEN^ins^ samples were compared using unpaired two-tailed t-test with Welch’s correction (* *p* < 0.05). All values shown are mean values from technical triplicates. (**G**) TMM adjusted CPM expression values (line at median) for *BCL2* and *BAX* in the cohort of 13 *PICALM::MLLT10*-rearranged acute leukemias. **H** Patient data of the 13 *PICALM::MLLT10*-rearranged acute leukemias. * indicates the patient from Ulm (PAT 1).

Consistent with this, we found remarkably high BCL-2 mRNA (Fig. 2A) and protein expression levels (Fig. 2B+C) as compared to other venetoclax-sensitive (VEN^sens^) or -insensitive (VEN^ins^) BCP-ALL cell lines and primary ALL patient-derived xenograft samples (PDX) (Fig. 2D). Primary chronic lymphocytic leukemia (CLL) samples were included as a prototypical BCL-2 dependent hematological malignancy that is particularly sensitive to venetoclax [11]. The leukemia cells of the patient also showed low protein expression of anti-apoptotic MCL-1 and intermediate expression of pro-apoptotic BAX (Fig. 2B+C).

Concurrently, intracellular protein staining revealed high BCL-2 expression, low BCL-XL and MCL-1 expression, and intermediate BAX expression (Fig. 2E), comparable to previously published data in VEN^sens^ and VEN^ins^ BCP-ALL PDX samples [8]. Using the expression levels of BCL-2, BCL-XL and MCL-1 from the published PDX cohort and our patient, we calculated the “mediators of apoptosis combinatorial score” (MAC-score), which reliably predicts response to 5-AZA/VEN in patients with AML [12].

Consistent with this published data for AML, VEN^sens^ BCP-ALL samples had significantly higher MAC-scores than VEN^ins^ samples, with the MAC-score of our patient ranking above the mean of VEN^sens^ samples (Fig. 2F).

Thus, the BCL-2^high^/MCL-1^low^/BAX^intermediate^ profile and high MAC-score of the patients’ leukemia cell represent a prototypic venetoclax-sensitive cell, which is concordant with the high ex vivo sensitivity and the excellent clinical response with rapid clearance of leukemic blasts and induction of molecular remission.

Apart from CLL and AML, venetoclax has demonstrated preclinical activity in T- and B-ALL models, including adult high-risk ALL. Clinical studies have reported response rates of up to 75% in newly diagnosed as well as relapsed or refractory ALL when venetoclax is combined with chemo- or immunotherapy, indicating its potential as a bridge to HSCT for eligible patients [13]. Additionally, recent studies reported sensitivity to BCL-2 inhibition in adult patients with *PICALM::MLLT10* + AL with miscellaneous immunophenotypes as well as in young patients with *PICALM::MLLT10* + AUL [6, 14].

Our patient’s excellent response to venetoclax after induction failure supports the hypothesis that *PICALM::MLLT10* + AL might represent a distinct subgroup, characterized by poor response to conventional chemotherapy but sensitivity to BCL-2 inhibition [7]. Consistent with this, we found high *BCL2* and *BAX* transcript expression levels in diagnostic samples of 12 additional pediatric *PICALM::MLLT10* + ALs (Fig. 2E+F, 16.88–1422.95, median 293.28 CPM). 50% of this group of patients exhibited a prednisone poor response (PPR; >1000 blasts/µl peripheral blood after the 7-day prednisone prephase of ALL induction chemotherapy), which has long been considered as a high-risk factor and most predictive for outcome [15], supporting the concept of a distinct HR subgroup of AL. The high expression of *BCL2* and *BAX* suggests that this otherwise refractory subgroup might display a particular sensitivity to targeted therapy with BH3 mimetics, i.e. BCL-2 bound BAX might act as a suicide bag in this particular type of leukemia.

To our knowledge, this is the first description of the successful induction of durable remission in a completely drug resistant, pediatric *PICALM::MLLT10* + BCP-ALL by venetoclax, which was identified by individual ex vivo DRP. Our report supports BCL-2 inhibition by venetoclax as a potential therapeutic option for *PICALM::MLLT10* + AL.

## Supplementary information

Supplemental Material and Methods

## Data Availability

The RNA sequencing data are available from the corresponding author (Klaus-michael.debatin@uniklinik-ulm.de) on reasonable request.
