# Supplementary material for: Remission induction in refractory, drug resistant pediatric PICALM::MLLT10+ B-cell acute lymphoblastic leukemia by venetoclax
Source: Leukemia. 2025 Apr 15;39(6):1520–5. doi: 10.1038/s41375-025-02591-w (PMC12133564; doi:10.1038/s41375-025-02591-w)
Supplement: Supplementary file 1 — Supplemental Material and Methods [file 41375_2025_2591_MOESM1_ESM.docx]

# Title

# Remission induction in refractory, drug resistant pediatric *PICALM::MLLT10*+ B-cell acute lymphoblastic leukemia by venetoclax

Alexandra Niedermayer^1,8*^, Jana Stursberg^1*^, Anke Katharina Bergmann^2,3^, Martin Zimmermann^4^, Gunnar Cario^5^, Monika Brüggemann^6^, Rolf Köhler^7^, Daniel Steinbach^1^, Christian Reimann^1^, Felix Seyfried^1^, Lüder Hinrich Meyer^1^, and Klaus-Michael Debatin^1,8, †^

^1^Department of Pediatrics and Adolescent Medicine, Ulm University Medical Center, Ulm, Germany

^2^Institute of Human Genetics, Hannover Medical School, Hannover, Germany

^3^Clinical Genetics and Genomic Medicine, University Hospital Würzburg, Würzburg, Germany

^4^Department of Pediatric Hematology/Oncology, Hannover Medical School, Hannover, Germany

^5^Clinic for Pediatric Oncology and Rheumatology (Children and Adolescent Medicine I), University Hospital Schleswig-Holstein, Kiel, Germany

^6^Internal Medicine II – Hematology and Oncology, University Hospital Schleswig-Holstein, Kiel, Germany

^7^Institute of Human Genetics, Heidelberg University Hospital, Heidelberg, Germany

^8^German Center for Child and Adolescent Health (DZKJ), partner site Ulm, Ulm, Germany *These authors contributed equally to this work.

^†^corresponding author: klaus-michael.debatin@uniklinik-ulm.de

# Supplemental Material

## **Material and Methods**

**Processing of blood and bone marrow samples**

Peripheral blood and bone marrow samples were collected from the patient at the indicated time points. Written informed consent was obtained in accordance with the Declaration of Helsinki and the institutional review board.

**Blast count**

Blast count was calculated based on the percentage of blasts and the total leukocyte count.

**Drug response profiling (DRP)**

Without co-culture:

Leukemic blasts isolated from the patient’s bone marrow at the end of induction therapy on day 33 were exposed to increasing concentrations of 24 anti-cancer drugs. Cell viability was determined after 24 hours by flow cytometry using forward/side scatter criteria on an Attune NxT (Thermo Fisher Scientific, Waltham, [Massachusetts,](https://www.google.com/search?sca_esv=b1ce1c954e88e9aa&q=Waltham&si=ACC90nyvvWro6QmnyY1IfSdgk5wwjB1r8BGd_IWRjXqmKPQqm7-ehbnP4YpijFxvceNm_UaAtnwFyKf3k4Cxe5AQUO4lYcOvU2Hn6b5GOoyNZlvRQWZFXsEKr6z0-mRIe4252nFMjjNLucavv2PoQdNEzlPJp5bt9-3gCWVKgX7bmvnEh2eKQdeyQl5vrf1CsP_Q2kHJfhvP&sa=X&sqi=2&ved=2ahUKEwiP9NqJ4uiJAxXw1wIHHYvAL2UQmxMoAHoECEIQAg) USA).

With hTERT MSC co-culture:

Primary bone marrow hTERT-immortalized mesenchymal stroma cells (MSCs) were kindly provided by Dario Campana. For co-culture experiments, MSCs were seeded on day -1 at 5x10^3^ per well in 96-well plates in RPMI-1640 medium supplemented with 20% fetal bovine serum, 1% L-Glutamine and 1% Penicillin/Streptomycin. After four hours, 1x10^5^ cryopreserved ALL cells from day 33 after induction therapy were stained with 1 µM CellTrace Violet (C34557, Thermo Fisher Scientific) and added to the MSC cultures in technical triplicates. On day 0, samples were exposed to increasing concentrations of the inhibitors for 24 hours. Cell viability was measured by flow cytometry (Attune NxT, Thermo Fisher Scientific, Waltham, [Massachusetts,](https://www.google.com/search?sca_esv=b1ce1c954e88e9aa&q=Waltham&si=ACC90nyvvWro6QmnyY1IfSdgk5wwjB1r8BGd_IWRjXqmKPQqm7-ehbnP4YpijFxvceNm_UaAtnwFyKf3k4Cxe5AQUO4lYcOvU2Hn6b5GOoyNZlvRQWZFXsEKr6z0-mRIe4252nFMjjNLucavv2PoQdNEzlPJp5bt9-3gCWVKgX7bmvnEh2eKQdeyQl5vrf1CsP_Q2kHJfhvP&sa=X&sqi=2&ved=2ahUKEwiP9NqJ4uiJAxXw1wIHHYvAL2UQmxMoAHoECEIQAg) USA) using forward/side scatter (FSC/SSC) criteria and CellTrace Violet staining to distinguish ALL cells from MSCs. Data analysis was performed using FlowJo 10.8 software.

**RNA sequencing**

**Patients:** Total RNA was isolated from mononucleated cells of the patients’ bone marrow at the time of primary diagnosis, as described previously [Suppl. 1]. The RNA-Seq library was prepared using the TruSight RNA Pan-Cancer Panel (Illumina, San Diego, California, USA) and was sequenced on an Illumina MiSeq. Fusion genes were identified using RNA-Seq alignment and TopHat Alignment in the BaseSpace RNA-Seq Alignment App by Illumina.

**BCP-ALL samples**: Total RNA was isolated from patient-derived xenograft samples. RNA-Seq library was prepared using the TruSeq RNA Sample Preparation Kit v2 (Illumina).and were sequenced on an Illumina NextSeq550.

**CLL samples**: RNA-Seq raw data from vehicle-treated CLL patient samples were used for analysis (GSE199037).

The expression browser in the CLC Genomics Workbench (Qiagen, version 23.0.5) was used to depict expression values of the samples as counts per million (CPM) after trimmed mean of M values (TMM) normalization.

The RNA sequencing data are available from the corresponding author (klaus-michael.debatin@uniklinik-ulm.de) on reasonable request.

**Immunoblotting**

Proteins were isolated using CHAPS lysis buffer with the addition of proteinase inhibitor. After lysates were incubated on ice for 30 min, supernatant was collected after centrifugation at 14,000 rpm for 30 min at 4 °C. Protein lysates were heated in sample buffer and reducing agent for 10 min at 70°C. Thereafter, proteins were separated by polyacrylamide gel electrophoresis, transferred to a nitrocellulose membrane and subjected to Western blotting. Immunoblots were developed using fluorescence and densitometric analysis was performed with ImageJ Software.

**Intracellular protein staining**

Cells were permeabilized (0.002% digitonin), fixed (formaldehyde), incubated in a

neutralizing buffer, and stained with rabbit anti-BCL-XL (Alexa Fluor 647, #86387, CST), rabbit anti-MCL-1 (Alexa Fluor 647, #78471, CST), mouse anti-BCL-2 (Alexa Fluor 488, #59422, CST), mouse anti-BAX (Alexa Flour 488, #633604, BioLegend), mouse IgG1 Isotype Control (Alexa Fluor 488, #4878, CST) and rabbit IgG Isotype Control (Alexa Fluor 647, #3452, CST) in triplicates. Samples

were measured (flow cytometry, Attune NxT, Thermo Fisher) and analyzed (FlowJo

10.7.1). MFIs were normalized to respective isotype controls.

# References

1. Schieck, M., Lentes, J., Thomay, K. *et al.* Implementation of RNA sequencing and array CGH in the diagnostic workflow of the AIEOP-BFM ALL 2017 trial on acute lymphoblastic leukemia. *Ann Hematol* **99**, 809–818 (2020). https://doi.org/10.1007/s00277-020-03953-3
